# Healthcare utilization in patients with spinal disorders referred for specialist evaluation in Norway: an observational register-based cohort study

**DOI:** 10.1186/s12913-026-15256-w

**Published:** 2026-08-03

**Authors:** Lise Grethe Kjønø, Kjersti Storheim, Kåre Rønn Richardsen, Bjørnar Berg, Marianne Bakke Johnsen, Stine Haugaard Clausen, Jan Hartvigsen, Margreth Grotle, Karin Magnusson

**Affiliations:** 1 Department of Rehabilitation Science and Health Technology, Faculty of Health Sciences, Oslo Metropolitan University, Oslo, Norway; 2 Department of Research and Innovation, Division of Clinical Neuroscience, Oslo University Hospital, Oslo, Norway; 3 Faculty of Health and Social Sciences, University of South-Eastern Norway, Drammen, Norway; 4 Department of Physical Medicine and Rehabilitation, Oslo University Hospital, Oslo, Norway; 5 Center for Muscle and Joint Health, Department of Sports Science and Clinical Biomechanics, University of Southern Denmark, Odense, Denmark; 6 Chiropractic Knowledge Hub, Odense, Denmark; 7 Cluster for Health Services Research, Norwegian Institute of Public Health, Oslo, Norway; 8 Clinical Epidemiology Unit, Department of Clinical Sciences, Orthopaedics, Faculty of Medicine, Lund University, Lund, Sweden

**Keywords:** Healthcare utilization, Spinal disorders, Register-based research

## Abstract

**Background:**

Healthcare utilization is high for people with spinal disorders. We aimed to investigate differences in recommended versus actual healthcare use among individuals with back & neck pain compared to individuals with isolated back or neck pain. Additionally, we aimed to examine variations in healthcare utilization patterns before and after a specialist evaluation based on the location of pain.

**Methods:**

We linked the Norwegian Neck and Back Registry with health registries, including patients with spinal disorders registered between January 1^st^, 2016, and December 31^st^, 2020 (9316 patients (63% with back pain, 27% with neck pain, 11% with back & neck pain) aged 18–70 years, mean (SD) age 44 (13) years, 57% females). We studied the percentages being recommended and actually visiting primary care physicians, physical therapists, and chiropractors in primary care and outpatient and inpatient consultations in specialist care after an outpatient specialist evaluation (index consultation), focusing on differences between groups based on the location of spinal pain.

**Results:**

In total, ~20%-80% followed healthcare recommendations. Patients with back & neck pain followed recommendations less often than patients with back pain (39% (95% CI 35.7, 41.8) vs 45% (95% CI 43.9, 46.4)) or neck pain (44% (95% CI 42.4, 46.3)). Participants with back & neck pain had a proportion of consultations with physical therapists at 29.7% (95% CI 25.1, 34.2) and 25.4% (95% CI 20.7, 30.1) in years 1 and 2 after index consultation, ~6 percentage points higher than the back pain group (1st: −5.8% (95% CI −10.6, −1.0), 2nd: −6.0% (95% CI −11.0, −0.9). They also had a 2 percentage point higher proportion of consultations with primary care physicians compared to the neck pain group (1st: −2.4% (95% CI −4.1, −0.8), 2nd: −1.5% (95% CI −2.7, −0.3)).

**Conclusions:**

Patients with mixed back & neck pain have lower adherence to recommended care and higher utilization of physical therapy and primary care physicians two years after specialist evaluation compared to patients with isolated spinal pain. These results suggest that the current approach to managing spinal pain should be reconsidered to better address the complex needs of this patient group.

**Supplementary Information:**

The online version contains supplementary material available at https://doi.org/10.1186/s12913-026-15256-w.

## Background

Spinal disorders, encompassing low back and neck pain, are the leading causes of years lived with disability across most countries and age groups [1, 2]. The prevalence and impact of spinal disorders have exhibited a significant surge over the recent decades, and from 1990 to 2015, the number of disability-adjusted life years (DALYs) attributed to low back and neck pain increased by 60% [3].

Spinal disorders are among the main reasons for seeking healthcare [4–6]. Research indicates that individuals with spinal disorders also tend to use healthcare services more frequently than the general population [7–9], both compared to persons without spinal disorders and to patients with other chronic musculoskeletal disorders, such as osteoarthritis, osteoporosis, and rheumatoid arthritis [10]. However, a large proportion of healthcare utilization is concentrated among a minority of the population [11]. For example, among patients with spinal pain, specific subgroups, typically older patients with comorbidities and poor overall health, use most healthcare services [12, 13]. Moreover, patients with spinal disorders evaluated in specialist care in Norway reported higher pain and lower health-related quality of life, and a greater proportion of this patient population had less education, a heavier workload, less physical activity and less reliance on social benefits than patients treated in primary care [14].

Less is known regarding variations in healthcare use *within* the patient population with spinal disorders, particularly among patients referred to specialist care and with different locations of pain. First, we have limited knowledge of what type of care these patients are being recommended by the specialist and to what extent recommended care is being followed [12]. For example, it may be hypothesized that patients with mixed back & neck pain receive different recommendations for follow-up care after a specialist evaluation than patients with isolated back or neck pain. When pain is more widespread, it may be more complex, which could be reflected in more specialist care use rather than primary care use, e.g., chiropractic or physical therapy. Knowledge of such patterns of healthcare use among patient groups defined by different pain locations is important for clinicians and health administrators to improve care quality, reduce disparities and use sparse resources as efficiently as possible [15, 16]. Second, we have limited knowledge of these patients’ healthcare use over a longer time frame. Healthcare utilization is influenced by a range of factors, such as higher pain intensity and disability, sociodemographic factors (age, gender), employment status, and beliefs about pain [17]. Still, there is a knowledge gap in patients with severe pain or spinal pain in more than one region. For example, a broad range, types and levels of healthcare use obtained from registry data and whether it differs by the location of pain has not previously been described in a group of patients with severe, long-lasting spinal pain referred for specialist evaluation [18].

Using a clinical quality registry covering specialist care for spinal disorders in Norway, we aimed to investigate differences in recommended versus actual healthcare use among individuals with back & neck pain compared to individuals with isolated back or neck pain. Additionally, we aimed to examine variations in healthcare utilization patterns before and after a specialist evaluation based on the location of pain, i.e. comparing individuals with more widespread spinal pain to individuals with more isolated pain in the back region vs neck region.

## Methods

### Study design, participants and setting

In this observational register-based cohort study, we included patients from the Norwegian Neck and Back Registry (NNRR), which is a clinical quality registry that includes patients with back and neck pain referred from primary healthcare to specialized multidisciplinary outpatient clinics in Norwegian public hospitals [19]. The NNRR was established in 2012 and expanded to include the four main university hospitals by 2016 and three additional hospitals by late 2020. The inclusion of the four major university hospitals covering different regions helps to ensure that the sample represents patients from each health region across the entire country. The coverage in the NNRR varied from 42% to 63% during these years (see Additional file 1 for details) [19]. The register includes patient-reported questionnaire data on demographics, lifestyle, pain, clinical outcomes, and Patient Related Outcome Measures (PROMs), along with data from healthcare provider-completed forms on clinical and radiological diagnosis, clinical examination findings and recommendations for further treatment pathways [19]. In our study, we included patients aged 18–70 years whose data were recorded between January 1^st^, 2016, and December 31^st^, 2020. If a patient had multiple records in the NNRR, we treated the first consultation date as the index consultation date.

The individual data from the NNRR were linked to data obtained from public healthcare registries in Norway for the period from 2008 to the end of 2021 using encrypted personal identification numbers (PINs), the Control and Payment of Health Refunds (KUHR) (primary care) and the Norwegian Patient Registry (NPR) (specialist care). KUHR encompasses reimbursement records from primary care physicians, physical therapists, chiropractors, and self-employed specialists contracted with regional health authorities [20]. NPR is a population-based national administrative health registry covering activity data from all specialist health services in Norway. It covers consultations and admissions in government-owned hospitals and outpatient clinics and visits to private contract specialists eligible for government reimbursement [21]. Primary care in Norway is organized by the municipalities and financed through the National Insurance Scheme, the municipalities, and patient co-payment [22]. Patients have direct access to primary care physicians, physical therapists (since 2018), and chiropractors. Co-payment rates vary among healthcare providers, with highest co-payment costs for chiropractic healthcare [22]. This study is part of the AID-Spine project, which has been approved by the ethics committee of the Health Region of South-East Norway (2022/371282), and the Data Protection Authority of Norway approved the registry protocol. All participants provided written informed consent upon participation in the NNRR, including consent to link their data to other registries.

### Pain locations

We used the International Classification of Disease (ICD-10) diagnostic codes recorded in the NNRR at the index consultation to categorize patients into three mutually exclusive pain location groups. Patients with back-related diagnostic codes, including mid-back pain, were grouped as “back pain”, patients with neck-related diagnostic codes as “neck pain”, and patients with both back and neck diagnostic codes were classified as “back & neck pain” (see Additional file 2 for details). In cases where the location could not be determined from the diagnostic code, the location of pain was determined using 1) other clinical variables specifying pain location from the healthcare provider form, 2) responses to the pain location question, or 3) scores derived from the Oswestry Disability Index (ODI) or Neck Disability Index (NDI) in the patient-reported questionnaire in the NNRR (detailed description and numbers in Additional file 1 and Additional file 3). Patients whose pain location could not be determined via these methods were excluded.

### Outcomes

In all analyses, we studied cause-specific healthcare use (consultations related to spinal disorders) across five healthcare services, as described in Additional file 4: 1) primary care physicians (including general practitioners, specialists under regional health authority contracts, and emergency wards), 2) primary care physical therapists (including physical therapists, manual therapists, and psychomotor physical therapists), and 3) chiropractors in primary care, 4) outpatient consultations and 5) inpatient consultations within specialist care. Inpatient consultations in specialist care included both day-patient consultations and overnight stays. A consultation was defined as a face-to-face or video healthcare encounter, excluding simple communication, prescription writing or administrative tasks.

### Statistics

To investigate differences in recommended versus actual healthcare use, we first analyzed descriptive data (age and sex) by pain location group and calculated the percentages (with 95% confidence intervals calculated based on Wilson) [23] of participants who were recommended for follow-up in primary care, specialist care, both primary- and specialist care, and no further follow-up. We investigated actual care by identifying patients’ first three consultations for spinal disorders within six months after the index consultation, categorizing them into the utilization of primary care, specialist care, both primary- and specialist care and no consultations. Furthermore, we investigated the use of recommended follow-up and categorized patients as either following or not following recommendations. Patients were considered to have followed recommendations if actual care aligned with the recommendations and if those advised to seek both primary and specialist care opted for only one.

For the examination of variations in healthcare utilization patterns before and after the specialist evaluation, we calculated proportions of participants (with 95% confidence intervals) who visited each of the five healthcare services at least once per month, from eight years before to two years after the index consultation, for participants in each of the pain location groups (back pain, neck pain or back & neck pain). The monthly percentages were then averaged over yearly quarters (3-month periods). Thus, healthcare use per quarter (months aggregated per quarter) was expressed as a binary outcome for each of the healthcare services: the outcome was set to 1 if the participant had at least one consultation during the quarter with the healthcare service in question and otherwise set to 0. Observations for the outcomes were censored from the quarter in which we could no longer observe healthcare use. The two-year follow-up period was chosen to balance the need for a sufficient sample size with the length of follow-up. As the quality of data in the NNRR improved significantly towards 2020, including patients up to this year allowed us to use the most reliable data while ensuring a robust sample size. These analyses were conducted for the five healthcare services and stratified by the three pain location groups. We included an interaction term between the pre-and post-periods and the subgroup category, allowing for the adjustment for time-invariant person-level confounders by design. To address background trends such as seasonal variations in healthcare use and the impacts of the COVID-19 pandemic, we incorporated calendar month fixed effects in our analyses [24]. This approach adjusts for external time variant trends that affect all groups similarly. Additionally, we adjusted for age and sex to account for potential confounding factors. Separate models were conducted for cause-specific healthcare utilization by each healthcare service as described above under outcomes.

We also present such averages for two periods following the index consultation: the first-year post-period (1–4 quarters after index consultation) and the second-year post-period (5–8 quarters after index consultation). Furthermore, for the first and second post periods, we assessed the difference in proportions of cause-specific healthcare use by pain location groups [24, 25]. For these analyses, the back & neck pain group was the group of interest, whereas the group with back pain vs the group with neck pain only served as reference categories in two separate analyses. The difference estimate, calculated using logistic regression, represents the difference in percentage points between the pain location groups. A zero difference estimate suggests no association between pain location groups and subsequent healthcare use [24]. We repeated all these analyses using all-cause healthcare utilization (consultations for any reason) to include healthcare use episodes not coded as a spinal diagnosis. We estimated 95% confidence intervals in all our models based on robust standard errors. All analyses were run using STATA MP v.17.

## Results

**Fig. 1:**
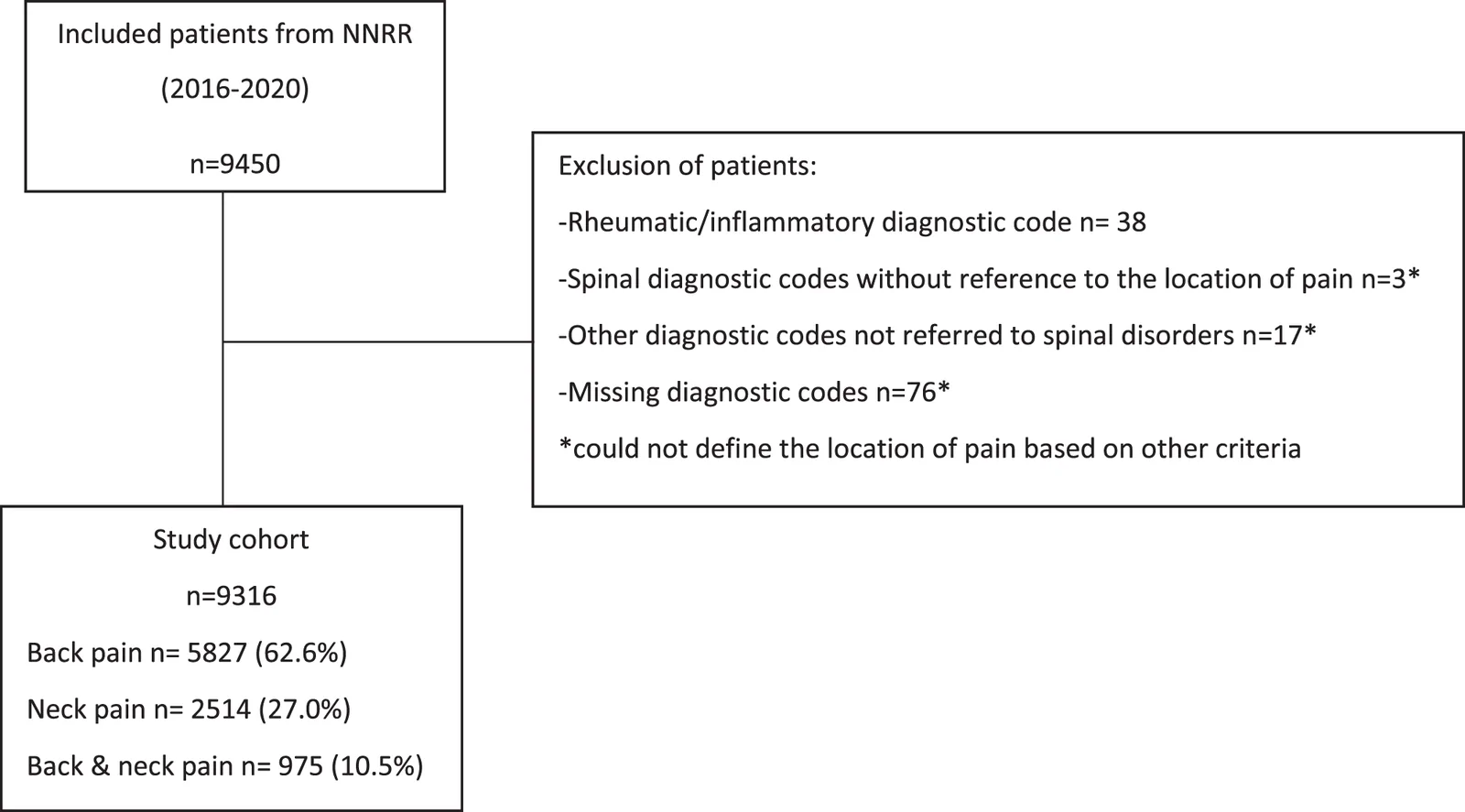
Flow chart of participants included in the study

Of the 9450 eligible participants from the NNRR, 9316 were included in the analysis (Fig. 1). The mean age (SD) was 44 (13) years; 57% were female, 63% had back pain, 27% had neck pain, and 11% had back & neck pain. Participants in the neck pain group were older and had a slightly higher proportion of females than those in the back pain group and the back & neck pain group (Table 1).

**Table 1:** Baseline characteristics and recommended follow-up by specialist after the index consultation

	All *n=*9316	Back pain *n=*5827 (63%)	Neck pain *n=*2514 (27%)	Back & neck pain *n=*975 (11%)
**Age,** mean (SD)	43.7 (13)	43.1 (13)	45.6 (12)	42.8 (13)
**Female**, n (%)	5 335 (57)	3 183 (55)	1 565 (62)	587 (60)
**Recommended follow-up** Percentage (95% CI)				
**Primary care** -physician* -physical therapist* -chiropractor* -other*	25.1 (24.3, 26.0) 13.5 (12.8, 14.2) 15.7 (15.0, 16.5) 0.6 (0.4, 0.7) 2.0 (1.8, 2.3)	23.2 (22.1, 24.3) 10.9 (10.1, 11.7) 15.5 (14.6, 16.4) 0.6 (0.5, 0.9) 1.8 (1.5, 2.2)	28.8 (27.0, 30.6) 16.8 (15.4, 18.3) 18.2 (16.8, 19.8) 0.5 (0.3, 0.9) 2.2 (1.7, 2.9)	27.7 (25.0, 30.6) 20.6 (18.2, 23.3) 10.8 (9.0, 12.9) 0.2 (0.1, 0.8) 2.9 (2.0, 4.1)
**Specialist care**	37.7 (36.7, 38.7)	39.6 (38.3, 40.8)	36.4 (34.6, 38.3)	29.5 (26.8, 32.5)
**Both primary & specialist care** -physician* -physical therapist* -chiropractor* -other*	21.3 (20.5, 22.2) 11.4 (10.8, 12.1) 12.1 (11.4, 12.8) 1.0 (0.9, 1.3) 1.8 (1.5, 2.1)	21.5 (20.5, 22.6) 10.6 (9.9, 11.4) 12.7 (11.8, 13.5) 1.2 (1.0, 1.5) 1.6 (1.3, 2.0)	22.7 (21.1, 24.4) 13.6 (12.3, 15.0) 12.0 (10.8, 13.3) 0.6 (0.4, 1.0) 2.4 (1.8, 3.0)	16.6 (14.4, 19.1) 10.8 (9.0, 12.9) 8.7 (7.1, 10.7) 1.0 (0.6, 1.9) 1.4 (0.9, 2.4)
**No follow-up**	15.9 (15.2, 16.6)	15.8 (14.9, 16.8)	12.1 (10.9, 13.4)	26.2 (23.5, 29.0)

*Categories may overlap internally

### Differences in recommended vs actual healthcare utilization

**Fig. 2:**
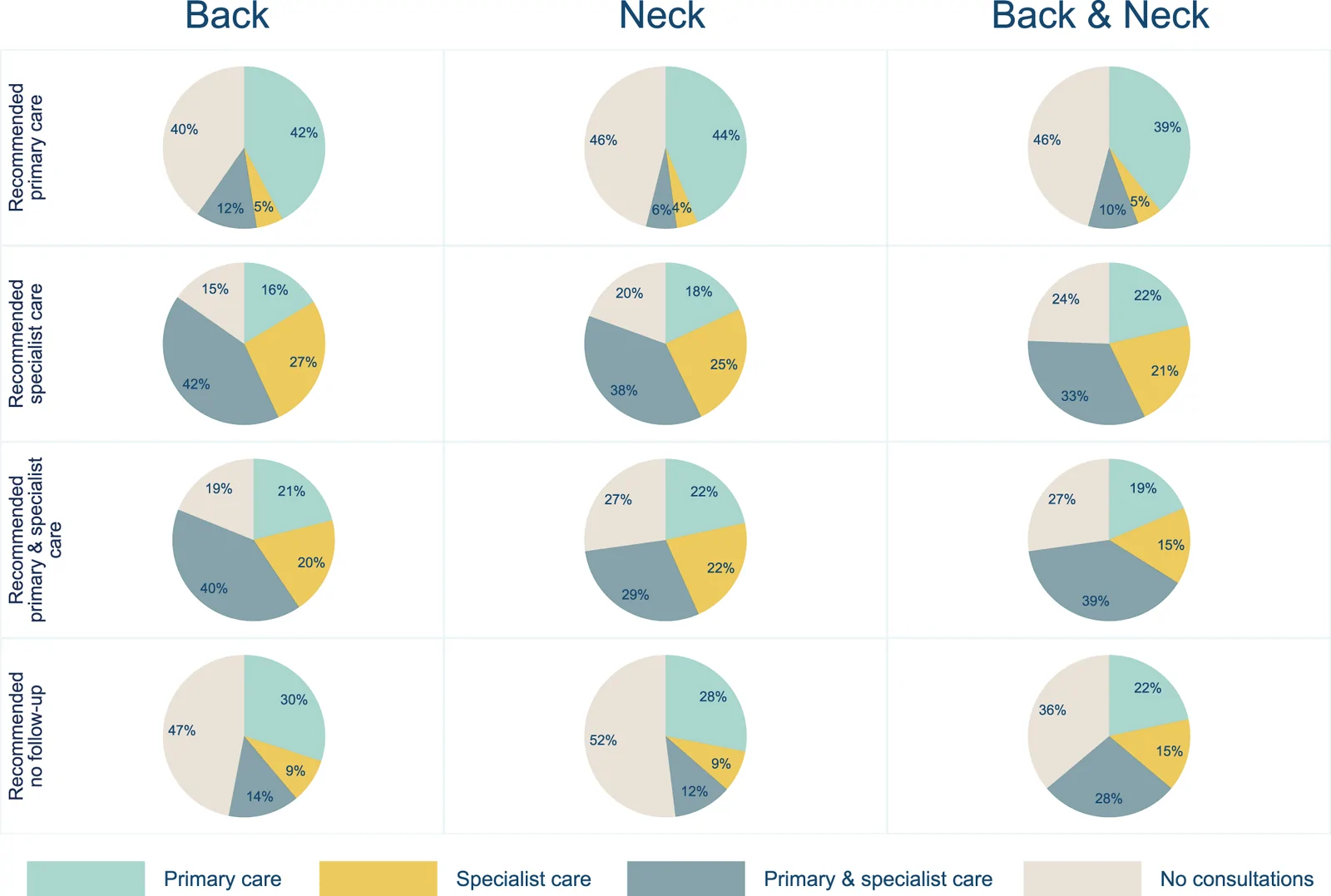
Variations in the use of primary and specialist healthcare according to recommendation

**Fig. 3:**
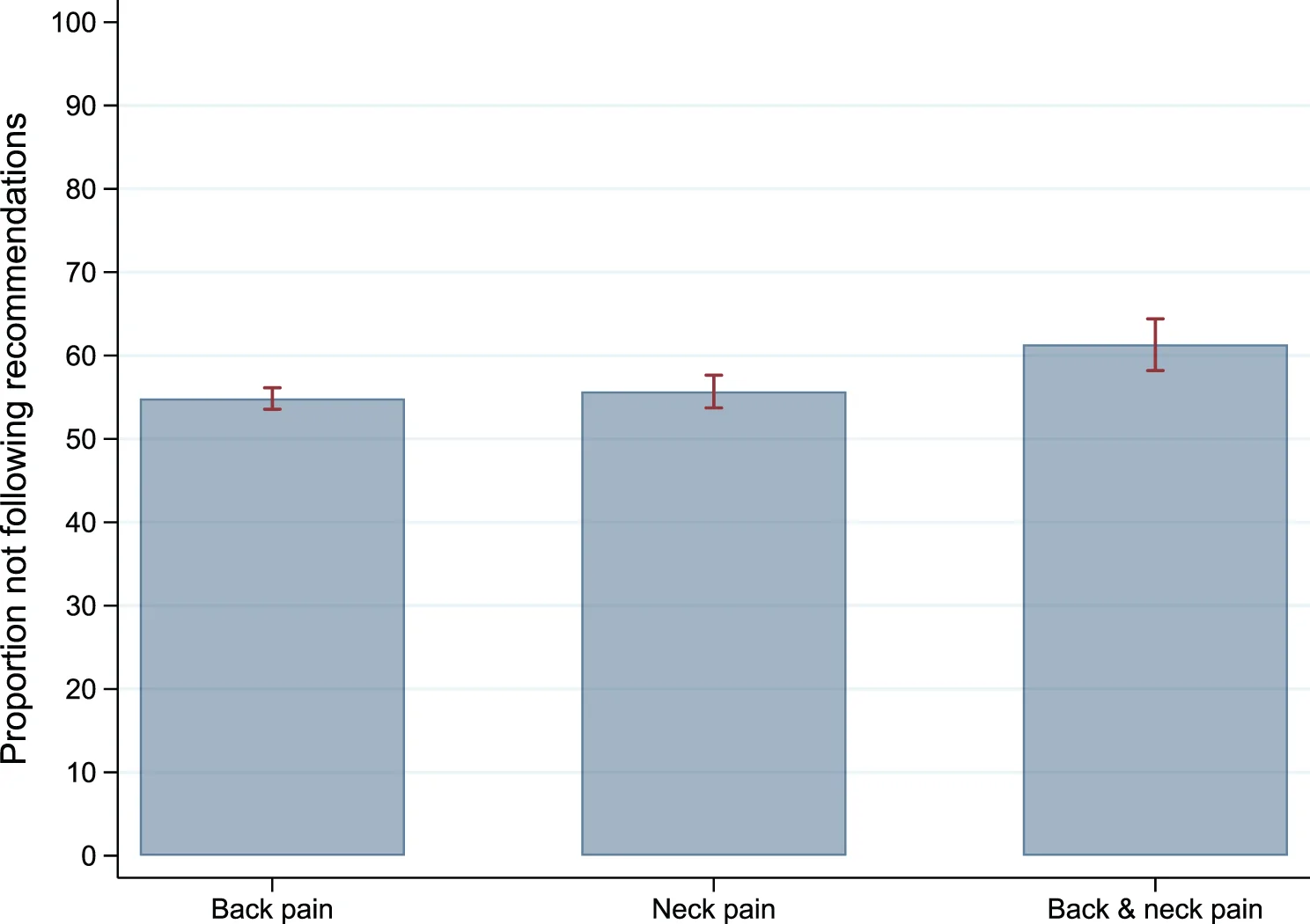
Proportions (95% CI) of patients who did not follow recommendations from the healthcare provider

At the index consultation, participants with back & neck pain, as well as participants with neck pain, were more often recommended for follow-up treatment in primary care than were those with back pain (28%, 29% versus 23%) (Table 1). Conversely, a higher proportion of participants with only back pain and only neck pain were recommended follow-up in specialist care than were those with mixed back & neck pain (40%, 36% versus 30%) (Table 1). Similar results were found for participants advised for follow-up in *both* primary and specialist care, with a higher proportion of individuals with isolated back pain and neck pain than those with mixed back & neck pain (22%, 23% versus 17%, respectively). Participants with back & neck pain had the highest proportion of those recommended no further follow-up after the index consultation (Table 1). Figure 2 shows the variations in primary and specialist care use, given the healthcare recommendations at the index consultation. In all pain location groups and for all healthcare services, ~20% to 80% followed the recommendations. Overall, patients with back & neck pain followed the recommended care slightly less often than those with back or neck pain (Fig. 3).

### Variations in healthcare utilization patterns before and after a specialist evaluation based on location of pain

#### Primary care physician utilization

**Fig. 4:**
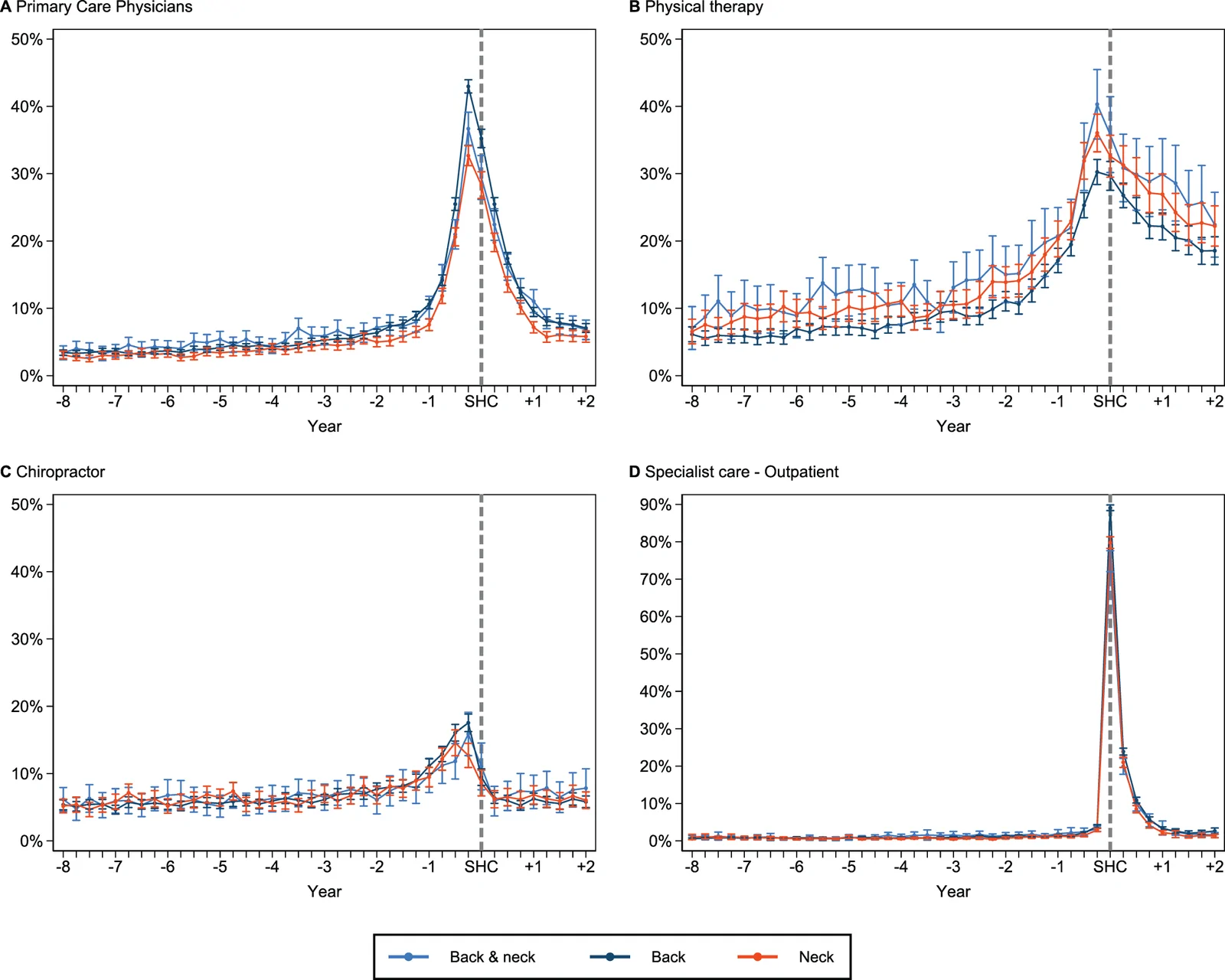
Cause-specific healthcare utilization across pain location groups and healthcare services. The y-axis shows the proportion of participants with at least one consultation per quarter. SHC: specialist healthcare (indicates the time point for index consultation)

The proportion of patients visiting primary care physicians at least once a quarter was relatively stable over time and stable across all pain location groups during the pre-period (Fig. 4A). In the first year following the index consultation, 15% of patients with back & neck pain visited primary care physicians, similar to those with back pain and ~2 percentage points higher proportion than those with neck pain (Table 2). During the second year, the proportion decreased to 7% but remained similar to that in the back pain group and was still ~2 percentage points higher than that in the neck pain group (Table 2). The latter implies, during the first two years after the index consultation, that for every 100 persons with back & neck pain, approximately two more persons visit a primary care physician at least once in a quarter for their complaints compared to those with only neck pain. Group differences were larger when analyzing all-cause primary care physician consultations, and the proportion of patients with back & neck pain visiting primary care physicians was consistently higher than that of patients with either back pain or neck pain alone (see Additional file 5 and Additional file 6).

**Table 2:** Differences in healthcare utilization between pain location groups

	First-year post period		Second-year post period	
	Percentage (95% CI)*	Percentage points difference (95% CI)*†	Percentage (95% CI)*	Percentage points difference (95% CI)*†
**Primary care**				
**Physicians**				
Back & neck	15.2 (13.8, 16.6)		7.4 (6.4, 8.4)	
Back	16.2 (15.6, 16.9)	1.0 (−0.5, 2.6)	7.6 (7.2, 8.1)	0.2 (−0.9, 1.3)
Neck	12.8 (12.0, 13.6)	−2.4 (−4.1, −0.8)	5.9 (5.3, 6.6)	−1.5 (−2.7, −0.3)
**Physical therapy**				
Back & neck	29.7 (25.1, 34.2)		25.4 (20.7, 30.1)	
Back	23.9 (22.4, 25.5)	−5.8 (−10.6, −1.0)	19.4 (17.7, 21.2)	−6.0 (−11.0, −0.9)
Neck	29.0 (26.5, 31.5)	−0.7 (−5.9, 4.5)	23.1 (20.5, 25.7)	−2.3 (−7.7, 3.1)
**Chiropractor**				
Back & neck	6.8 (4.8, 8.8)		7.3 (5.3, 9.4)	
Back	6.0 (5.4, 6.5)	−0.8 (−2.9, 1.3)	5.8 (5.2, 6.4)	−1.5 (−3.7, 0.7)
Neck	6.5 (5.6, 7.5)	−0.3 (−2.5, 2.0)	6.3 (5.3, 7.3)	−1.0 (−3.3, 1.3)
**Specialist care**				
**Outpatient**				
Back & neck	9.4 (8.2, 10.7)		1.8 (1.3, 2.7)	
Back	11.2 (10.7, 11.7)	1.8 (0.4, 3.1)	2.4 (1.9, 2.9)	0.6 (−0.1, 1.3)
Neck	9.2 (8.6, 9.8)	−0.2 (−1.6, 1.2)	1.5 (1.1, 1.9)	−0.3 (−1.0, 0.3)
**Inpatient**				
Back & neck	1.8 (0.9, 2.7)		1.0 (0.1, 2.0)	
Back	1.8 (1.5, 2.2)	0.0 (−0.9, 1.0)	0.9 (0.6, 1.3)	−0.1 (−1.1, 0.9)
Neck	1.9 (1.2, 2.6)	0.1 (−1.5, 0.4)	0.5 (0.2, 0.8)	−0.6 (−1.5, 0.4)

The table presents data on cause-specific consultations across all pain location groups and healthcare services

*The percentage (95% CI) of participants in the pain location groups, adjusted for age, sex, and calendar month

†Difference estimates (in percentage points) represent the mean values (95% CI) from quarterly analyses within the respective time periods using back pain vs neck pain as reference groups in separate analyses

#### Physical therapy utilization

Over the eight years before the index consultation, the proportion of patients with back & neck pain utilizing physical therapy was higher than those with neck pain, and the lowest proportion was seen in patients with back pain (Fig. 4B). In the first year following the index consultation, 30% of patients with mixed back & neck pain visited physical therapists at least once per quarter, decreasing to 25% in the second year. This was ~6 percentage points higher than the back pain group and similar to those in the neck pain group for both years, as shown in Table 2. The difference estimates imply that during the first two years after the index consultation, for every 100 persons with back & neck pain, around six more persons would visit a physical therapist at least once in a quarter for their complaints compared to those with only back pain. Analyses of all-cause healthcare utilization to physical therapists showed a similar pattern to cause-specific consultations. However, there were approximately 10–15% higher proportions of all-cause healthcare use throughout the period, with difference estimates showing a larger difference between participants with back & neck pain compared to those with back pain, as shown in additional files (see Additional file 5 and Additional file 6).

#### Chiropractor utilization

The percentage of patients who consulted chiropractors remained at 5–8% per quarter across all pain location groups eight to two years before the index consultation, with an increase to 13–17% in the quarter before the index consultation (Fig. 4C). In the first and second years following the index consultation, 7% of the patients with back & neck pain sought chiropractic care. This was similar to prior levels, and no differences were observed between those with either back or neck pain (Table 2). Analyses of all-cause chiropractor utilization revealed similar results, showing no differences between the pain location groups after the index consultation (Additional file 6) and only marginally higher consultation rates throughout the entire period (Additional file 5).

#### Outpatient utilization

In the years preceding the index consultation, only 1–3% of patients across pain location groups visited outpatient services for spinal disorders quarterly (Fig. 4D). During the first quarter after the index consultation, the average proportion of cause-specific outpatient consultations rose to 22% before decreasing to approximately 10% during the second quarter (Fig. 4D). The proportion of patients with back & neck pain who sought outpatient services in the first year following the index consultation was 9% (Table 2). This number was lower than that in the back pain group and similar to that in the neck pain group (Table 2). During the second year after the index consultation, the proportion returned to approximately similar to that observed before the index consultation (Fig. 4D), at 2% for the back & neck pain group, with no difference to the back or neck pain groups (Table 2). In terms of all-cause outpatient utilization, 42 and 30% of the participants in the back & neck pain group used outpatient services in the first and second years following the index consultation, respectively. There were no group differences to the back or neck pain groups (Additional file 6).

#### Inpatient utilization

Across pain location groups, the proportion of inpatient healthcare use remained below 1% per quarter in cause-specific analyses throughout the pre-period (Additional file 7). However, there was a notable increase following the index consultation. In the first year after the index consultation, 2% of patients with back & neck pain received inpatient healthcare, decreasing to 1% during the second year of follow-up (Table 2). The difference estimates suggested no group differences in the utilization of inpatient consultations for spinal disorders (Table 2). The proportions were slightly higher in the analysis of all-cause healthcare utilization (Additional file 5). However, these estimates were similar between the groups (Additional file 6).

## Discussion

Among 9316 patients referred to specialist evaluation due to spinal disorders, we found that the recommendation for further healthcare after a specialist evaluation varied by pain location. Patients with back & neck pain, as well as those with only neck pain, were more often recommended for follow-up in primary care (28 and 29% versus 23% for only back pain). Conversely, those with only back or only neck pain were more often recommended follow-up in specialist care (40 and 36% versus 30% for mixed pain). Additionally, patients with mixed pain had the highest proportion of no further follow-up recommendations. Furthermore, patients followed these recommendations differently depending on pain location, with only 21% of those with back & neck pain who were recommended for follow-up in specialist care, whereas 81% with back pain who were recommended for follow-up in both primary & specialist care. The group with mixed back & neck pain had a 6-percentage point higher proportion of consultations with physical therapists in primary care 1–2 years after the index consultation compared to patients with back pain alone. Additionally, these patients had a 2-percentage point higher proportion of consultations with primary care physicians compared to patients with neck pain during the same period.

### Strengths and limitations

This study is the first to explore healthcare utilization among patients with spinal disorders referred for specialist evaluation using the NNRR linked with health registries. Important strengths of our study are the sample size and use of routinely collected data from health registries, which are legally mandated and cover the entire population of Norway. By using a panel data approach following individuals over time, we could adjust for person-level confounders by design and adjust for seasonal variations in healthcare use, and the impacts of the COVID-19 pandemic.

The main limitation of this study is the coverage rate in the NNRR. Although 15 outpatient clinics were eligible for inclusion in the register in 2020, only four clinics delivered data to the NNRR in 2016, with three additional clinics joining in 2020 [19]. From 2016 to 2020, the coverage rates at the individual level for these included outpatient clinics were 48%, 56%, 63%, 56% and 42%, respectively. This low coverage rate can be attributed to the NNRR being a relatively new quality registry, with some larger hospitals encountering technical challenges in scanning patient-reported forms into the register. The lowest coverage rate was observed in 2020, which was mainly impacted by challenges related to form completion during the COVID-19 pandemic [19]. The establishment of the NNRR has been an ongoing process involving the incorporation of potential outpatient clinics and overcoming challenges related to data scanning. Another related limitation is the potential lack of representativeness of our sample. While the presence of outpatient clinics from the four largest hospitals in Norway suggests representation across each health region, we lack specific data to directly compare our cohort to the entire population of patients referred to specialists for spinal disorders in Norway. This limits our ability to thoroughly assess representativeness. Therefore, our findings must be considered in light of the data used in our analyses being represented only in the initial stages of this registry’s operation.

Additionally, we lacked data on patients referred for specialist evaluation by general practitioners but who were subsequently rejected by the outpatient specialist. In that regard, our findings should be considered in light of the conditioning on visiting a specialist, i.e., all patients had to survive up until the index consultation, which could introduce selection bias and, therefore, influence the interpretation of our findings. For example, we could not prospectively follow a group of patients with spinal disorders from several years before specialist evaluation and compare them to a control group of patients with spinal disorders who were not referred for specialist evaluation. This would have provided a more representative picture of care use both before and after a specialist visit. Another limitation is the potential underestimation of healthcare utilization, as the public registers do not include data from private healthcare services.

Regarding diagnostic coding within the NNRR, individuals with missing or non-spine-related diagnostic codes (13%) were categorized based on self-reported pain location, which was predominantly observed among individuals in the back & neck pain group. Self-reporting may lead to increased registrations of mixed pain, diverging from physician-assigned diagnostic codes, potentially resulting in more individuals being registered with both back and neck pain. Additionally, there is a risk of not accurately capturing all primary care consultations, especially in cause-specific analyses, as general practitioner visits often address multiple problems, increasing diagnostic accuracy uncertainty [26]. However, we used the main- and bi-diagnoses from KUHR, which likely enhanced our ability to capture the range of issues addressed during consultations. Lastly, our study primarily focused on clinical encounters, like visits to healthcare providers in primary and specialist care. Unfortunately, our data sources did not include data on the relative escalation of care, such as diagnostic imaging, laboratory tests, or interventional procedures. Including such aspects could provide a more comprehensive understanding of healthcare utilization patterns. We propose this as a topic for future research.

### Interpretation and comparison with previous studies

To our knowledge, this study is novel among cohorts of patients with spinal disorders referred for outpatient specialist evaluations. This limits direct comparisons with existing literature. However, our results align with population studies indicating higher healthcare utilization among patients with mixed symptoms than those with isolated back or neck pain. Concerning healthcare use following specialist evaluation, a Dutch cohort study investigating characteristics and treatment relationships in patients referred to a secondary spine center found that most patients were indicated for nonsurgical treatments [27]. Specifically, 83% of patients received care outside the hospital (including physical therapy, general practitioner services, and expectant management), 32% received secondary care treatments (by pain specialist, rehabilitation, referral to another specialist, and use of corset), and 25% did not receive any further treatment or follow-up [27]. This study revealed a lower proportion of patients receiving follow-up in specialist care and a higher proportion of patients receiving primary care than observed in the present study. However, the data in the Dutch study were collected at the initial outpatient visit, providing information solely on allocated treatments. Therefore, their findings can only be compared to the present study with respect to recommended treatments. Utilizing national registries enabled us to investigate actual healthcare utilization following specialist evaluation, revealing a large gap between recommended and actual care, with adherence proportions ranging from 21% to 81%. Notably, we found that patients with mixed back & neck pain were particularly less likely to follow the recommendations from the specialist. Several factors may contribute to this disparity. First, the complexity of managing multiple pain conditions presents challenges for patients in navigating treatment options and consistently following recommendations, as comprehensive care is often required [28, 29]. Second, demographic or poor psychosocial characteristics, such as age, socioeconomic status, or mental health challenges like stress, depression, or anxiety, may negatively affect adherence [30, 31]. Third, healthcare system factors, such as access to healthcare services, availability of specialists, and communication between providers and patients, can impact adherence [32]. Patients with mixed pain might face barriers such as longer wait times for specialist appointments or difficulty in coordinating care among multiple providers. Moreover, patients’ perceptions of treatment effectiveness might influence their adherence. If patients with mixed back & neck pain do not perceive significant relief or improvement from the recommended care, they may be less likely to follow through with the treatment plan. The implications of these adherence disparities may be considerable, as poor adherence can result in worse treatment outcomes, increased healthcare utilization, and higher rates of chronic pain and disability. However, the extent to which adherence to recommendations affects outcomes in this patient group is not well understood. Hence, future research should explore the impact of adherence on outcomes for these patients, considering the complexity of their conditions and the barriers they face.

Analyses of all-cause consultations revealed a more pronounced disparity between the back & neck pain group and participants with isolated back or neck pain, particularly for consultations with primary care physicians (Additional file 5). This suggests that patients with back & neck pain have more complex healthcare needs than patients with back or neck pain, extending beyond spinal disorders. Additionally, this discrepancy may also indicate that patients with missing diagnostic codes in the NNRR likely have primary pain issues not directly related to spinal complaints, with a higher prevalence of additional complaints beyond spinal disorders. This finding raises the possibility that individuals in the back & neck pain group may present a higher prevalence of comorbidities compared to those in the back pain and neck pain groups, indicating a more complex clinical presentation. Thus, our data support previous findings suggesting that spinal disorders are often associated with comorbidities [33], and patients experiencing a mix of back and neck pain exhibit more comorbidities compared to those with back pain or neck pain only [34]. Furthermore, comorbidities are linked to increased healthcare utilization [35–37]. This finding aligns with our findings of higher healthcare use among patients with back & neck pain. Together, the current and previous studies highlight the need for healthcare providers to consider the multifaceted nature of their conditions and tailor treatment strategies accordingly, addressing not only spinal issues but also potential comorbidities and associated symptoms.

## Conclusion

In total, between ~20 and 80% followed the healthcare recommendations by the specialist, depending on spinal pain location. Our findings highlight that the recommendation of care type and level, as well as the proportion of healthcare utilization after a specialist evaluation, varies by pain location: Patients with mixed back & neck pain followed the recommended care less often than patients with only back pain or neck pain. They also had slightly higher use of physical therapy and consultations with primary care physicians in the follow-up period.

## Electronic supplementary material

Below is the link to the electronic supplementary material.

Supplementary Material 1

## Data Availability

The datasets used in the current study are based on quality registry and national registries and are not publicly available. Access to pseudonymized data is only granted through application to the Norwegian Centre for Research Data and Regional Committees for Medical and Health Research Ethics. The corresponding author can provide other data and materials upon reasonable request.

## Abbreviations

ICD-10: International Classification of Diseases
KUHR: The Control and Payment of Health Refunds
NNRR: Norwegian Neck and Back Registry
NPR: Norwegian Patient Registry
SHC: Specialist health care
