# Supplementary material for: Healthcare utilization in patients with spinal disorders referred for specialist evaluation in Norway: an observational register-based cohort study
Source: BMC Health Serv Res. 2026 Aug 3;26:1508. doi: 10.1186/s12913-026-15256-w (PMC13726017; doi:10.1186/s12913-026-15256-w)
Supplement: Supplementary file 1 [file 12913_2026_15256_MOESM1_ESM.docx]

# Supplementary Information

## **Additional file 1: Methods**

### Coverage rate

The inclusion of clinics in the NNRR has been ongoing since the registry was established in 2012 (1). Since 2016, outpatient clinics at the four largest hospitals in Oslo, Bergen, Trondheim, and Tromsø have provided data for the NNRR. In 2020, three more outpatient clinics affiliated with the NNRR contributed data for parts of the year. Moreover, 15 other outpatient clinics were identified as potential contributors to the registry, although they had not yet commenced data contributions. Outpatient clinics not affiliated with the NNRR were excluded from coverage calculations. From 2016 to 2020, the national coverage rates in the NNRR were 48%, 56%, 63%, 56% and 42%, respectively. From 2016 to 2019, the coverage rate was determined by averaging the respective coverage rates of the outpatient clinics included (1). In 2020, the coverage rate was calculated by dividing the total number of registrations by the total number of relevant patients in the included outpatient clinics. Technical difficulties in scanning patient-reported forms partially explained the lower coverage rate observed. Additionally, the reduced coverage in 2020 was influenced by challenges facilitating form completion due to the COVID-19 pandemic, especially at the largest hospitals (1).

### Categorization into location of pain groups

Other clinical variables specifying pain location from the healthcare provider form were variables indicating whether radiculopathy radiated from the back or neck and whether unspecific pain was related to the back or neck. The question “Where is your pain?” involves checking for the body regions that have experienced pain for the last 14 days and covers 16 body regions on the front side and 16 body regions on the back side of the body. Patients were asked to identify and indicate any areas within the regions where they had experienced pain. Patients who selected “low back” and “chest back (upper part of the back)” on the back side of the body were categorized into the “back pain” group. Patients who indicated “neck” on the back side or front side of the body were classified into the “neck pain group.” Patients who were categorized into both the “back pain” and “neck pain” groups based on the pain question, were classified into the “back & neck pain” group. When the location of pain was still lacking, categorization employed the Oswestry Disability Index (ODI) for “back pain” and the Neck Disability Index (NDI) for “neck pain”. Patients who reported both scores were classified as having “back & neck pain”.

### Outcomes

Patients were classified into mutually exclusive groups based on the following recommendations for further treatment provided by healthcare providers at the index consultation: 1) follow-up in primary care, 2) follow-up in specialist care, 3) follow-up in both primary and specialist care, or 4) no further follow-up. Recommended follow-up in primary care included consultations with physicians, physical therapists, chiropractors, and others (including psychologists, municipal follow-up, or work-related follow-up). Specialist care follow-up included treatment in one's specialist service, referral to another rehabilitation centre, referral for evaluation by other specialists, or assessment for surgical intervention. This encompassed individual and group multidisciplinary interventions, ranging from simple follow-ups to comprehensive treatments. We investigated actual care by identifying patients’ first, second, and third consultations within six months after the index consultation. Consultations with physicians, physical therapists, and chiropractors were considered in primary care, while all outpatient and inpatient consultations were categorized as specialist care. Patients were subsequently classified into the following groups: primary care, specialist care, both primary and specialist care, or no follow-up.

**Additional file 2:** Diagnostic codes with categorization into back pain and neck pain.

| Diagnostic codes | Term |
| --- | --- |
| Back pain |  |
| M413 | Thoracogenic scoliosis |
| M4195 | Scoliosis, thoracolumbar region |
| M4215 | Adult osteochondrosis of spine, thoracolumbar region |
| M4306 | Spondylolysis, lumbar region |
| M4316 | Spondylolisthesis, lumbar region |
| M4317 | Spondylolisthesis, lumbosacral region |
| M4726 | Other spondylosis with radiculopathy, lumbar region |
| M4728 | Other spondylosis with radiculopathy, sacral and sacrococcygeal region |
| M4784 | Other spondylosis, thoracic region |
| M4786 | Other spondylosis, lumbar region |
| M4787 | Other spondylosis, lumbosacral region |
| M4806 | Spinal stenosis, lumbar region |
| M4854 | Collapsed vertebra, not elsewhere classified, thoracic region |
| M510 | Thoracic, thoracolumbar and lumbosacral intervertebral disc disorders with myelopathy |
| M511 | Thoracic, thoracolumbar and lumbosacral intervertebral disc disorders with radiculopathy |
| M512 | Other thoracic, thoracolumbar and lumbosacral intervertebral disc displacement |
| M513 | Other thoracic, thoracolumbar and lumbosacral intervertebral disc degeneration |
| M518 | Other thoracic, thoracolumbar and lumbosacral intervertebral disc disorders |
| M519 | Unspecified thoracic, thoracolumbar and lumbosacral intervertebral disc disorder |
| M533 | Sacrococcygeal disorders, not elsewhere classified |
| M5385 | Other specified dorsopathies, thoracolumbar region |
| M5416 | Radiculopathy, lumbar region |
| M5417 | Radiculopathy, lumbosacral region |
| M543 | Sciatica |
| M544 | Lumbago with sciatica |
| M5446 | Lumbago with sciatica, lumbar region |
| M545 | Low back pain |
| M5456 | Low back pain, lumbar region |
| M5457 | Low back pain, lumbosacral region |
| M5458 | Low back pain, sacral and sacrococcygeal region |
| M546 | Pain in thoracic spine |
| M5464 | Pain in thoracic spine, thoracic region |
| M5468 | Pain in thoracic spine, sacral and sacrococcygeal region |
| M548 | Other dorsalgia |
| M5485 | Other dorsalgia, thoracolumbar region |
| M5486 | Other dorsalgia, lumbar region |
| M5487 | Other dorsalgia, lumbosacral region |
| M549 | Dorsalgia, unspecified |
| M5494 | Dorsalgia, thoracic region |
| M5495 | Dorsalgia, thoracolumbar region |
| M5496 | Dorsalgia, lumbar region |
| M5497 | Dorsalgia, lumbosacral region |
| M5498 | Dorsalgia, sacral and sacrococcygeal region |
| G834 | Cauda equina syndrome |
| *Ryggplager | Low back pain |
| *Thorakale ryggsmerter | Pain in thoracic spine |
| *Uspesifikke ryggsmerter | Low back pain |
|  |  |
| Neck pain | |
| M4302 | Spondylolysis, cervical region |
| M4712 | Other spondylosis with myelopathy, cervical region |
| M4722 | Other spondylosis with radiculopathy, cervical region |
| M4782 | Other spondylosis, cervical region |
| M4792 | Spondylosis, cervical region |
| M4802 | Spinal stenosis, cervical region |
| M500 | Cervical disc disorder with myelopathy |
| M501 | Cervical disc disorder with radiculopathy |
| M503 | Other cervical disc degeneration |
| M509 | Cervical disc disorder, unspecified |
| M530 | Cervicocranial syndrome |
| M531 | Cervicobrachial syndrome |
| M5392 | Dorsopathy, cervical region |
| M5412 | Radiculopathy, cervical region |
| M5413 | Radiculopathy, cervicothoracic region |
| M542 | Cervicalgia |
| M5421 | Cervicalgia, occipito-atlanto-axial region |
| M5422 | Cervicalgia, cervical region |
| M5423 | Cervicalgia, cervicothoracic region |
| *Nakke | Cervicalgia |
| *Nakkeplager | Cervicalgia |
| *Nakkesmerter | Cervicalgia |
| *Uspesifikke nakkeplager | Cervicalgia |
| *Diffuse nakkeplager | Cervicalgia |
| *Generaliserte nakkeplager | Cervicalgia |
|  |  |
| Spinal diagnostic codes without reference to the location of pain | |
| M411 | Juvenile and adolescent idiopathic scoliosis |
| M418 | Other forms of scoliosis |
| M419 | Scoliosis, unspecified |
| M420 | Juvenile osteochondrosis of spine |
| M430 | Spondylolysis |
| M431 | Spondylolisthesis |
| M471 | Other spondylosis with myelopathy |
| M472 | Other spondylosis with radiculopathy |
| M478 | Other spondylosis |
| M480 | Spinal stenosis |
| M540 | Panniculitis affecting regions of neck and back |
| M541 | Radiculopathy |
| *Skoliose | Scoliosis |

The same person may have more than one diagnostic code. *Diagnoses written with the term in the diagnosis variable are included because the location of pain is defined.

**Additional file 3:** Number of participants categorized into pain location groups based on criteria for categorization

|  | **ICD-10 codes** | **ICD-10 codes+ diagnostic variables** | **ICD-10 codes + diagnostic variables + pain question** | **ICD-10 codes + diagnostic variables + pain question + ODI/NDI** |
| --- | --- | --- | --- | --- |
| **Back, n (%)** | 5 174 (54.8) | 5 409 (57.2) | 5 616 (59.4) | 5 827 (61.9) |
| **Neck, n (%)** | 2 349 (24.9) | 2 420 (25.6) | 2 506 (26.5) | 2 514 (26.7) |
| **Back & Neck, n (%)** | 412 (4.4) | 412 (4.4) | 683 (7.2) | 975 (10.4) |
| **Spinal diagnostic codes without reference to location of pain, n (%)** | 257 (2.7) | 68 (0.7) | 17 (0.2) | 3 (0.03) |
| **Other diagnoses, n (%)** | 294 (3.1) | 232 (2.5) | 45 (0.5) | 17 (0.2) |
| **Missing, n (%)** | 926 (9.8) | 871 (9.2) | 545 (5.8) | 76 (0.8) |

**Additional file 4:** Diagnostic codes used to define cause-specific consultations from KUHR and NPR

| **Data source** | **Back** | **Neck** |
| --- | --- | --- |
| **KUHR (ICPC)** | L02 L03 L84 L85 L86 | L01 L83 |
| **NPR (ICD-10)** | G544 G551 G552 G553 G558 G834 M400/M405 M412 M415 M418/M419 M430/M432 M435 M438/M439 M471/M472 M478/M484 M488 M489 M498 M510/M514 M518/M519 M532/M533 M538 M539 M541 M543/M546 M548/M549 M960/M961 M963/M964 M966 M968/M969 M991 M993/M997 | G999 M500/M503 M508/M509 M530/M531 |

**Additional file 5:** All-cause healthcare utilization across pain location groups and healthcare services.

SHC: Specialist healthcare indicates the time point for index consultation.

The y-axis shows the proportion of participants with at least one consultation per quarter.

**Additional file 6:** Differences in healthcare utilization between pain location groups

|  | **First-year post period** | | **Second-year post period** | |
| --- | --- | --- | --- | --- |
|  | **Percentage (95% CI)*** | **Percentage points difference (95% CI)*†** | **Percentage (95% CI)*** | **Percentage points difference (95% CI)*†** |
| **Primary care** |  |  |  |  |
| **Physicians** |  |  |  |  |
| Back & neck | 48.8 (46.7, 50.9) |  | 42.6 (40.3, 44.9) |  |
| Back | 43.0 (42.2, 43.9) | -5.8 (-8.0, -3.6) | 36.6 (35.8, 37.5) | -6.0 (-8.4, -3.5) |
| Neck | 43.5 (42.3, 44.7) | -5.3 (-7.7, -2.9) | 36.1 (34.9, 37.4) | -6.5 (-9.1, -3.9) |
| **Physical therapy** |  |  |  |  |
| Back & neck | 43.9 (39.8, 48.1) |  | 40.7 (36.3, 45.2) |  |
| Back | 34.8 (33.2, 36.4) | -9.1 (-13.5, -4.6) | 32.1 (30.3, 33.9) | -8.6 (-13.4, -3.9) |
| Neck | 44.1 (41.8, 46.4) | 0.2 (-4.6, 4.9) | 39.3 (36.6, 42.0) | -1.4 (-6.6, 3.8) |
| **Chiropractor** |  |  |  |  |
| Back & neck | 8.6 (6.4, 10.8) |  | 9.0 (6.8, 11.2) |  |
| Back | 6.9 (6.3, 7.5) | -1.7 (-4.1, 0.6) | 6.8 (6.1, 7.5) | -2.2 (-4.5, 0.1) |
| Neck | 7.9 (6.9, 8.9) | -0.7 (-3.2, 1.7) | 7.8 (6.7, 8.9) | -1.2 (-3.7, 1.3) |
| **Specialist care** |  |  |  |  |
| **Outpatient** |  |  |  |  |
| Back & neck | 42.4 (40.0, 44.8) |  | 29.7 (26.7, 32.7) |  |
| Back | 40.6 (39.7, 41.5) | -1.8 (-4.3, 0.8) | 25.7 (24.5, 26.8) | -4.0 (-7.3, -0.8) |
| Neck | 41.0 (39.6, 42.4) | -1.4 (-4.2, 1.4) | 26.3 (24.5, 28.1) | -3.4 (-6.9, 0.0) |
| **Inpatient** |  |  |  |  |
| Back & neck | 6.2 (5.0, 7.5) |  | 5.9 (4.5, 7.3) |  |
| Back | 5.4 (4.9, 5.8) | -0.8 (-2.2, 0.5) | 5.0 (4.4, 5.6) | -0.9 (-2.4, 0.7) |
| Neck | 5.9 (5.1, 6.8) | -0.3 (-1.8, 1.2) | 4.1 (3.5, 4.7) | -1.8 (-3.3, -0.3) |

The table presents data on all-cause consultations across all pain location groups and healthcare services.

*The percentage (95% CI) of participants in the pain location groups, adjusted for age, sex, and calendar month.

†Difference estimates (in percentage points) represent the mean values (95% CI) from quarterly analyses within the respective time periods using back pain vs neck pain as reference groups in separate analyses.

**Additional file 7:** Cause-specific inpatient healthcare utilization across pain location groups

References

1.	The Norwegian Neck and Back Registry 2024 [updated 14.03.2024; cited 02.01.2025. Available from: <https://www.unn.no/fag-og-forskning/medisinske-kvalitetsregistre/norsk-nakke-og-ryggregister>.
